# Detection of antimicrobial resistance in *Glaesserella parasuis* in South China using whole-genome sequencing

**DOI:** 10.3389/fmicb.2024.1532743

**Published:** 2025-01-07

**Authors:** Qianwen Ge, Liangxing Fang, Yang Yu, Ruanyang Sun, Xiaoping Liao, Peng Zhang

**Affiliations:** ^1^National Risk Assessment Laboratory for Antimicrobial Resistance of Animal Original Bacteria, South China Agricultural University, Guangzhou, China; ^2^National Reference Laboratory of Veterinary Drug Residues, Department of Veterinary Pharmacology, College of Veterinary Medicine, South China Agricultural University, Guangzhou, China; ^3^Guangdong Provincial Key Laboratory of Veterinary Pharmaceutics Development and Safety Evaluation, South China Agricultural University, Guangzhou, China

**Keywords:** *Glaesserella parasuis*, antimicrobial resistance, whole-genome sequencing, phenotypic resistance, genotypic resistance

## Abstract

**Introduction:**

*Glaesserella parasuis* causes Glässer’s disease in pigs, a leading cause of death in swine herds and a major contributor to economic losses in the global swine industry. Although several studies have investigated antimicrobial resistance in *G. parasuis*, the correlation between phenotypic and genotypic resistance remains unclear due to incomplete genetic resistance mechanisms detection.

**Methods:**

The susceptibility of 117 clinical *G. parasuis* isolates to 7 antimicrobials was determined using a broth microdilution method. The sequences of 48 resistant isolates were obtained by whole-genome sequencing. Resistance genes, mutations, and group 1 *vtaA*s were detected based on whole-genome sequence data. Sequence types (STs) were identified by multilocus sequence typing (MLST).

**Results:**

Phenotypic analysis showed that most isolates were susceptible to the tested antibiotics; resistance was most common against tetracycline (27%) and enrofloxacin (20%). All isolates were susceptible to ceftiofur. Analysis of whole-genome sequences revealed that resistance to tetracycline, amoxicillin, erythromycin, florfenicol, and chloramphenicol was frequently associated with the resistance genes *tet*(B) or *tet*(H), *bla*_*ROB–1*_, *erm*(T), *floR*, and *catA3*, and enrofloxacin resistance was associated with mutations in GyrA, ParC, and ParE. MLST identified 25 STs, of which, 14 were novel. The sequenced strains were divided into two primary lineages, LI and LII. Group 1 *vtaA* genes were detected in 87.5% (*n* = 42) of the isolates.

**Conclusion:**

This study provides comprehensive insights into the molecular mechanisms responsible for drug resistance in *G. parasuis*, the characteristics of molecular epidemiology, and the virulence of resistant groups. Our findings can aid in the development of *G. parasuis*-specific clinical breakpoints and inform strategies for managing antimicrobial resistance in swine herds.

## 1 Introduction

*Glaesserella parasuis* commonly colonizes the upper respiratory tract of pigs and can cause Glässer’s disease in immunocompromised hosts (Zhang et al., 2019; Costa-Hurtado et al., 2020). Discovering virulence factors is key to understanding the pathogenesis of *G. parasuis*. Several putative virulence-associated genes have been reported, such as those encoding capsule, lipopolysaccharides, trimeric autotransporters, and outer membrane proteins, among others. Among them, virulence-associated trimeric autotransporter (*vtaA*) genes are the most important virulence factors, as some of the group 1 *vtaA*s are more frequently used in diagnostic settings to predict the virulence potential of strains (Mugabi et al., 2023). Certain sequence types (STs) of *G. parasuis* isolates are responsible for outbreaks of infections (Mugabi et al., 2023). Multilocus sequence typing (MLST) has achieved high levels of discrimination and is useful for understanding the evolution and epidemiology of *G. parasuis*.

Antibiotics are widely administered to animals to treat bacterial infections, prevent diseases, and promote growth (Kimera et al., 2020). However, the overuse and misuse of antimicrobial agents in animals contribute to the growing threat of antimicrobial resistance (AMR) (Mudenda et al., 2023). Under the pressure of antimicrobial selection, antimicrobial-resistant bacterial strains acquire a greater ability to proliferate in animals, reducing the effectiveness of antimicrobial drugs used in animal-derived food production (Velazquez-Meza et al., 2022). Continuous monitoring of AMR is essential to address this threat and can help guide empirical treatment recommendations (Gulumbe et al., 2022). Antimicrobial susceptibility testing and polymerase chain reaction (PCR) analysis are primary methods for routine phenotypic and genotypic resistance monitoring. While PCR may not detect all resistance mechanisms, whole-genome sequencing (WGS) provides comprehensive insights into known resistance-related genes and mutations in resistant strains (Yang et al., 2023).

In China, *G. parasuis* strains remain susceptible to most antibiotics; however, an increasing trend of AMR is observed (Zhang et al., 2019). Most studies have focused on the phenotypic susceptibility of *G. parasuis* isolates (Zhou et al., 2010; Brogden et al., 2018). Few studies have used the traditional PCR method to determine the genes or mutations responsible for phenotypic resistance (Dayao D. et al., 2016). As WGS has become more affordable in recent years, it has been used to detect resistance elements in various bacteria (McDermott et al., 2016; Bossé et al., 2017; Fen et al., 2023). In a recent study, we applied WGS to gain genomic insights into the diversity of *G. parasuis* strains found worldwide (Gong et al., 2024). However, without antimicrobial susceptibility data, resistance mechanisms cannot be linked to phenotypic resistance. Therefore, we primarily aimed to obtain comprehensive information on the determinants of resistance in resistant *G. parasuis* isolates. The susceptibility of 117 *G. parasuis* isolates to 7 antimicrobial compounds was tested in this study. Resistant isolates were screened for associated resistance mechanisms using WGS. Moreover, we also characterized the molecular epidemiology and virulence of the resistant isolates.

## 2 Materials and methods

### 2.1 Bacterial isolates, culture conditions, and identification

A total of 117 *G. parasuis* isolates, supplied by the National Reference Laboratory of Veterinary Drug Residues (Guangzhou, China), were isolated from pigs with Glässer’s disease in South China from 2010 to 2017. Isolates were cultured on tryptic soy agar supplemented with 10 μg/ml of nicotinamide adenine dinucleotide and 5% (v/v) fetal calf serum for 18 h at 37°C. Bacterial species were identified using 16S rRNA diagnostic PCR as described previously (Oliveira et al., 2001).

### 2.2 Antimicrobial susceptibility testing

*In vitro* antimicrobial susceptibility testing was performed using the broth microdilution method, as described previously (Prüller et al., 2017). The following antimicrobial agents and test ranges were used: amoxicillin (AMX, 0.12–256 μg/ml), ceftiofur (CEF, 0.12–128 μg/ml), enrofloxacin (ENR, 0.12–128 μg/ml), erythromycin (ERY, 0.25–256 μg/ml), florfenicol (FFC, 0.12–128 μg/ml), tetracycline (TET, 0.12–128 μg/ml), and chloramphenicol (CHL, 0.5–128 μg/ml). Quality control was performed using *Escherichia coli* ATCC 25922 and *Staphylococcus aureus* ATCC 29213. As resistance breakpoints specific to *G. parasuis* have not been reported, the breakpoints used in this study were derived from previous studies when available (Zhou et al., 2010). For AMX, if the minimum inhibitory concentrations (MICs) showed a bimodal distribution, isolates with high MICs were considered “resistant” in this study.

### 2.3 WGS and draft genome sequence assembly

*G. parasuis* isolates that were resistant to at least one of the tested antibiotics were subjected to WGS. Briefly, colonies were scraped from tryptic soy agar plates supplemented with 10 μg/ml nicotinamide adenine dinucleotide and 5% (v/v) fetal calf serum, which were cultured at 37°C for 18 h. Genomic DNA was extracted using the TIANamp Bacteria DNA Kit (Tiangen, Beijing, China) following the manufacturer’s instructions. WGS was performed with 250-bp reads on the MiSeq platform (Illumina, San Diego, CA, USA). Trimmed reads were assembled using SPAdes v3.6.2 (Bankevich et al., 2012).

### 2.4 Phylogenetic analysis

Single nucleotide polymorphism (SNP) phylogenetic analysis was performed using Parsnp v1.7.4,^1^ and the conserved regions were then filtered out with Pandas v2.0.3 using in-house scripts. A core-genome SNP phylogenetic tree was generated and then visualized using the online tool iTOL.^2^

### 2.5 Multilocus sequence typing analysis

Sequence types (STs) were determined by querying the assembled sequences using the *G. parasuis* pubMLST typing platform.^3^ Novel alleles and STs were assigned by submitting the data to the pubMLST *G. parasuis* database. The strains were divided into different groups according to their allelic profiles using BURST analysis of PubMLST plugins.^4^ A clone complex (CC) was defined if these strains contained at least five identical alleles; otherwise, the STs were considered singletons.

### 2.6 Identification of resistance genes and mutations

Acquired antimicrobial resistance genes were identified using ResFinder v4.6.0^5^ with default settings. The threshold for %ID was 90% and the minimum length was 60%. The 16-membered macrolides resistance gene *est*T was not present in the database; therefore, we downloaded its reference sequence for *Sphingobacterium faecium* strain WB1 plasmid pWB1 (CP094932.1) from NCBI. Chromosomal mutations associated with AMR were determined in *gyrA*, *gyrB*, *parC*, *parE*, *rplD* (L4), *rplV* (L22), and 23S rRNA using online BLAST software^6^ (Piddock, 2002; Eaves et al., 2004). *G. parasuis* Nagasaki (CP018034.1) was used as the reference strain.

### 2.7 Detection of group 1 *vtaA*s

The 406-bp amplicon sequence of Group1 *vtaA*s was extracted from the complete sequence of *G. parasuis* strain Nagasaki (CP018034.1) using the primers reported in a previous study. Blastn was used to screen for *vtaA*s in the assembled sequences by querying the 406-bp amplicon.

## 3 Results

### 3.1 Phylogenetic analysis, multilocus sequence types, and virulence

**FIGURE 1:**
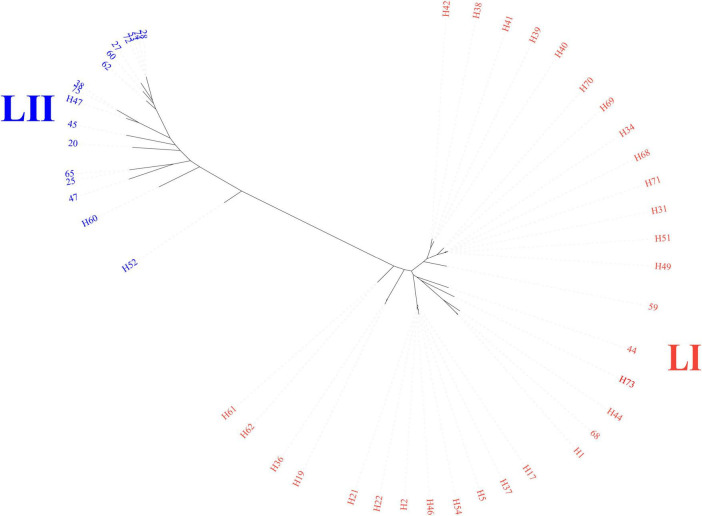
Core-genome phylogenetic tree of 48 sequenced genomes. Color shades indicate the primary lineages; LI and LII.

The core-genome SNP phylogenetic tree showed high genetic diversity and was divided into two primary lineages (LI and LII) based on the structure of the phylogenetic tree (Figure 1). The lineage of LI contained 31 isolates, larger than LII, which contained 17 isolates.

**FIGURE 2:**
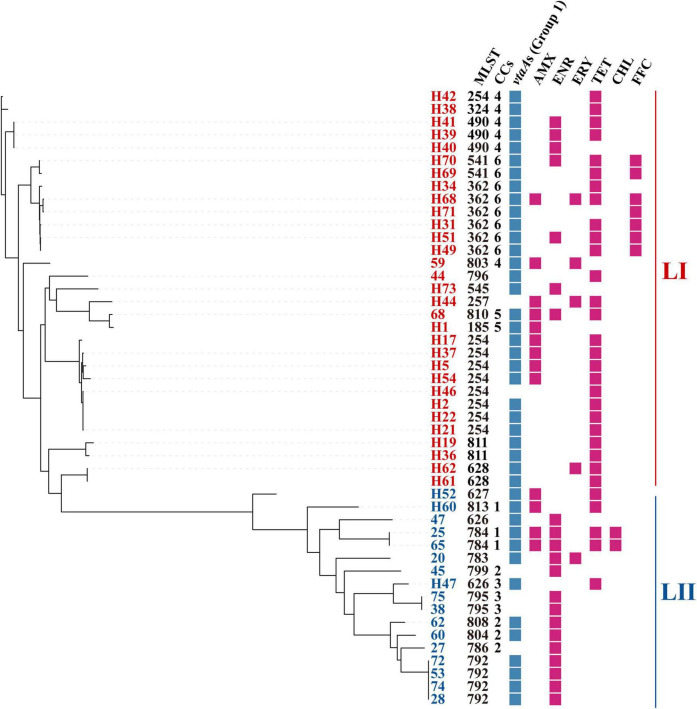
Distribution of STs, CCs, group 1 *vtaA*s, and phenotypic resistance. Core-genome phylogenetic tree was constructed based on core-genome SNPs. STs, sequence types; CCs, clonal complexes; AMX, amoxicillin; ENR, enrofloxacin; ERY, erythromycin; TET, tetracycline; CHL, chloramphenicol. FFC, florfenicol.

A total of 25 STs were identified within the collection of 48 sequenced isolates, among which, 14 were novel (Figure 2). The most common STs were ST254 (*n* = 8) and ST362 (*n* = 6), associated with LI. ST792 (*n* = 4) was the dominant ST within LII, attributed to clonal dissemination according to core genome analysis. More than half of the isolates (*n* = 26) were divided into 6 CCs, and the others belonged to 22 singletons. The most prevalent CC (CC6) included 2 sequence types, ST362 and ST541, and was part of LI.

Group 1 *vtaA*s were identified in 87.5% (*n* = 42) of the isolates. In detail, 93.5% (*n* = 29) of the LI isolates were positive for group 1 *vtaA*s, but only 76.4% (*n* = 13) of the LII isolates contained group 1 *vtaA*s (Figure 2).

### 3.2 Phenotypic resistance

Each of the 117 *G. parasuis* isolates was tested for susceptibility to seven antimicrobial agents. Among these isolates, 41% (*n* = 48) displayed resistance to at least one of the tested antibiotics. The most common resistances were to TET (27%, *n* = 27), ENR (20%, *n* = 23), and AMX (11%, *n* = 13), followed by FFC (6%, *n* = 7), ERY (4%, *n* = 5), and CHL (2%, *n* = 2). All 117 isolates were susceptible to CEF (Table 1). No correlation between phenotypic resistance and STs, lineages, or virulence factors was found in this study (Figure 2).

**TABLE 1:** MIC distribution of 117 *Glaesserella parasuis* isolates.

Antimicrobial agents	No. of isolates with MIC values (μ g/ml) of											
	**0.12**	**0.25**	**0.5**	**1**	**2**	**4**	**8**	**16**	**32**	**64**	**128**	**256**
Amoxicillin	4*	3	14	15	43	25						13*
Ceftiofur	79*	37	1									
Enrofloxacin	41*	11	6	36	6	4	13					
Erythromycin		27*	13	33	23	16		1		4		
Tetracycline	13*	4	51	14	3	1	4	26	1			
Florfenicol	41*	26	29	8	6		5	2				
Chloramphenicol			99*	9		7		2				

*Number of isolates with MICs equal to or higher/lower than concentrations of the test range. The resistance breakpoints are indicated by black vertical lines when available. MIC, minimum inhibitory concentration.

Among the 117 *G. parasuis* isolates, 59% (*n* = 69) showed no resistance to any of the tested antimicrobial agents and 6.8% (*n* = 8) displayed resistance to at least three antibiotics. The most common resistance phenotype was ENR (*n* = 13), followed by TET (*n* = 11), AMX+TET (*n* = 6), ENR+TET (*n* = 3), TET+FFC (*n* = 3), and AMX+ENR+TET+CHL (*n* = 3) (Table 2).

**TABLE 2:** Antimicrobial resistance profiles of 117 *Glaesserella parasuis* isolates.

No. of antibiotics	Resistance profile (no. of isolates)	Total no. of isolates (%)
0		69 (59%)
1	ENR (13) TET (11) AMX (1) FFC (1)	26 (22%)
2	AMX+TET (6) ENR+TET (3) TET+FFC (3) AMX+ERY (1) ENR+ERY (1)	14 (12%)
3	ENR+TET+FFC (2) AMX+ERY+TET (1) ENR+ERY+TET (1)	4 (3%)
4	AMX+ENR+TET+CHL (3) AMX+ERY+TET+FFC (1)	4 (3%)

ENR, enrofloxacin; TET, tetracycline; AMX, amoxicillin; FFC, florfenicol; CHL, chloramphenicol; ERY, erythromycin.

### 3.3 Genotypic resistance

A total of 12 known resistance genes and 6 mutations were identified in our WGS isolates, which predicted AMR to the 7 tested antibiotics and additional drugs (*sul2* for sulfonamide, *strA* and *strB* for streptomycin, *aph(3*′*)-Ic* for gentamicin, and *lnu*(C) for lincomycin). Among these resistance genes and mutations, 12 were associated with a decreased susceptibility to the tested antimicrobial compounds. Of the 48 resistant isolates evaluated using WGS, 31 were resistant to TET and harbored *tet*(B) (*n* = 27) or *tet*(H) (*n* = 2) (Table 3). Nineteen isolates were susceptible to TET and were negative for *tet* alleles. However, two isolates were susceptible to TET positive for *tet*(B) and had MICs of 2 and 4 μg/ml.

**TABLE 3:** Resistance phenotype and genotype of 48 sequenced *Glaesserella parasuis* isolates.

Antimicrobial agents	No. isolates with R or S phenotype	Resistance gene(s) or mutation(s) (no. isolates)
Amoxicillin	R, 13	*bla*_ROB–1_ (*n* = 13)
	S, 35	None
Ceftiofur	R, 0	None
	S, 48	None
Erythromycin	R, 5	*erm*(T) (*n* = 2) *erm*(T) + L4 (T10N, S13A) (*n* = 1) A2059G + L4 (T10N, S13A) (*n* = 1)
	S, 43	L4 (T10N, S13A) (*n* = 8) L4 (G42S) (*n* = 1) L22 (A93G) (*n* = 1) L22 (G91D) (*n* = 1) L4 (K63T) + L22 (A95V) (*n* = 1)
Tetracycline	R, 31	*tet*(B) (*n* = 29), *tet*(H) (*n* = 2)
	S, 17	*tet*(B) (*n* = 2)
Chloramphenicol	R, 2	*catA3* (*n* = 2) *floR* (*n* = 7)
	S, 46	None
Florfenicol	R, 7	*floR* (*n* = 7)
	S, 41	None

R, resistant; S, susceptible.

No acquired genes associated with quinolone resistance were detected among the sequenced isolates, whereas genomes of all *G. parasuis* isolates contained at least one mutation within the quinolone resistance-determining regions (QRDRs) of GyrA, ParC, and ParE (Table 4). Mutations were absent in GyrB. Fourteen of the twenty-three resistant isolates contained two mutations in GyrA (Ser83 and Asp87) and mutations in the QRDRs of ParC and ParE. Three resistant isolates contained two mutations at the Ser83 and Asp87 codons of GyrA and a Glu84Lys substitution in ParC. Isolates containing only one substitution at Ser83 of GyrA, with or without mutations in ParC and ParE, were susceptible to ENR. Substitutions at codons Ser83 and Asp87 combined with additional mutations in ParC were found in both resistant and susceptible isolates.

**TABLE 4:** MICs for enrofloxacin and resistance mutations in QRDRs of 48 sequenced *Glaesserella parasuis* isolates.

MIC (μ g/ml)	No. of isolates	Mutations within QRDRs		
		**GyrA**	**ParC**	**ParE**
8	6	S83Y, D87N	S80I	S458F
	1	S83F, D87Y	S80I	S458F
	1	S83F, D87N	S80I	S458F
	1	S83F, D87N	S80R	S458F
	2	S83F, D87Y	E84K	
	1	S83F, D87N	E84K	
	1	S83F, D87N	S80R	
4	2	S83F, D87N	S80I	S458A, H518Y
	1	S83F, D87Y	S80R	H518Y
	1	S83F, D87N	S80R	
2	2	S83F, D87Y	S80R	H518Y
	1	S83F, D87N	S80R	
	3	S83Y, D87H	S80R	
1	1	S83F		H518Y
	1	S83F, D87H	S80R	
	1	S83F, D87N	S80R	
	1	S83Y, D87H	S80R	
0.5	1	S83Y, D87H	S80R	
	1	S83Y, D87H	S80R	
≤ 0.12	7	S83F, D87H	S80R	
	3	S83Y, D87H	S80R	
	3	S83F, D87N	S80R	
	1	S83Y	S80R	H518Y
	1	S83Y	S80I	N516K
	1	S83Y	S80R	H518Y
	1	S83F		S458A, H518Y
	2	S83F		

QRDRs, quinolone resistance-determining regions; MIC, minimum inhibitory concentration.

Four of the erythromycin-resistant isolates, contained known macrolide resistance genes or mutations, with MICs of 64 μg/ml (Table 3). Three resistant isolates harbored *erm*(T), which encodes a methyltransferase that confers macrolide resistance. One isolate contained the mutation A2059G in the 23S rRNA gene. All genomes were also screened for mutations in the L4 and L22 proteins, encoded by the *rplD* and *rplV* genes. Thirteen sequenced isolates harbored at least one mutation in L4 or L22. These mutations were identified in both the susceptible and resistant isolates.

Each AMX-resistant isolate harbored *bla*_ROB–1_, which encodes a beta-lactamase. Seven isolates were resistant to FFC, all harboring the resistance-associated *floR* gene. Only two isolates were resistant to CHL and contained the *catA3* resistance gene. Notably, isolates with CHL MICs of 4 μg/ml contained the *floR* gene (Table 3).

## 4 Discussion

*G. parasuis* infections are common in modern swine production, resulting in economic losses. To control infections, antimicrobial treatment is one of the most important approaches. As antimicrobials were widely used as feed additives in China to promote growth before 2020, AMR in various bacteria, including *G. parasuis*, is increasing (Zhang et al., 2019; Shao et al., 2021). Therefore, the rise of AMR in *G. parasuis* is a growing concern, and the characterization of phenotypic and complete genotypic AMR represents a vital strategy for eliminating resistant strains.

This study sequenced 48 resistant *G. parasuis* isolates using WGS. Phylogenetic analysis indicated 2 primary lineages, LI and LII. This is similar to another study conducted in China, which was different from that reported in other countries, which showed that *G. parasuis* isolates were clustered into 3 primary lineages (Wan et al., 2020; Mugabi et al., 2023).

MLST is now integrated with WGS, which contributes to our understanding of the evolution and molecular epidemiology of *G. parasuis*. We identified 25 STs within the sequenced isolates, of which 14 were novel. In addition, almost half of the isolates had singleton STs, highlighting the heterogeneity and instability of *G. parasuis* strains. Other studies have also observed the diverse nature of *G. parasuis* (Wang et al., 2016; Turni et al., 2018; Gong et al., 2024). Most of the isolates with identical STs showed a close evolutionary relationship in our study, suggesting clonal dissemination of *G. parasuis* in certain farms and areas, which was reported in previous studies (Wu et al., 2023).

Several putative virulence genes are associated with the pathogenicity of *G. parasuis*. However, detection of all the putative virulence genes might lead to confusing results, as over half were conserved in *G. parasuis*. Group 1 *vtaA*s were considered good predictors of virulence, and some of them had been experimentally verified based on previous research (Olvera et al., 2012). In the current study, we chose group 1 *vtaA*s as the only virulence marker and screened them through our collection. Group 1 *vtaA*s were identified in most of the isolates in this study, which might be explained by the fact that all the strains were isolated from diseased pigs. In the present study, a larger number of LI isolates were observed containing group 1 *vtaA*s compared with those in LII. Mugabi et al. (2023) also found that LI and LII isolates carried a higher number of group 1 *vtaA* genes on average than LIII isolates.

Most *G. parasuis* isolates were susceptible to the tested antimicrobials. Only 7% of the isolates were resistant to at least three antibiotics. Our study confirms the high prevalence of antimicrobial-susceptible strains (Dayao et al., 2014). As antimicrobials such as gentamycin, streptomycin, and the combination of trimethoprim/sulphamethoxazole were not tested in this study, the frequency of multidrug resistance was lower than that previously reported in China (13%) (Zhou et al., 2010).

We observed a high level of TET resistance (27%), which was similar to that reported in another study (29%) (Zhang et al., 2019). TET resistance was predominantly conferred by *tet*(B) (27/29) and less commonly by *tet*(H) (2/29) in the present study. In a global investigation of *G. parasuis*, two TET resistance determinants, *tet*(B) and *tet*(H), have been detected, with the dominant *tet* allele being *tet*(B) (Lancashire et al., 2005; Gong et al., 2024). Two isolates that contained *tet*(B) had MICs that did not reach the resistance breakpoint. This finding suggests that a TET resistance breakpoint or an intermediate MIC cut-off specific to *G. parasuis* may be established.

We identified many ENR-resistant *G. parasuis* isolates as reported in other studies (Guo et al., 2011). Quinolone resistance was usually conferred by QRDR mutations and/or plasmid-mediated quinolone resistance genes. In this study, isolates possessing multiple mutations (two in GyrA, one in ParC, and others in ParE) within the QRDRs were resistant to ENR. However, a single substitution of Ser83 in GyrA, along with other QRDR mutations in ParC and/or ParE did not confer ENR resistance. Two *G. parasuis* isolates with a single mutation in GyrA exhibit moderate resistance to ENR (Guo et al., 2011). These two isolates contain plasmid-mediated quinolone resistance genes [*qnrB6* or *aac(6*′*)-Ib-cr*]. No substitutions were observed in the GyrB protein of all the sequenced isolates.

According to the Annual Report on the Development of Veterinary Medicine Industry in China (for 2013 and 2014), doxycycline and enrofloxacin have become two of the four leading antimicrobial agents used in veterinary medicine, which might explain the high prevalence of TET- and ENR-resistant *G. parasuis* isolates.

Five *G. parasuis* isolates showed resistance to ERY. The frequency of ERY-resistant isolates in the present study was similar to that in another study in China in 2010 (Zhou et al., 2010) but considerably lower than that in a recent study (Zhang et al., 2019). These results suggest an increasing macrolide resistance in *G. parasuis*. Several studies have reported susceptibility data for macrolides; however, reports on the underlying resistance mechanisms are less common. The resistance gene *erm*(T) and the A2059G mutation in 23S rRNA are responsible for macrolide resistance in *G. parasuis* (Dayao D. A. E. et al., 2016). In the present study, we found that three resistant isolates contained the *erm*(T) gene, and one resistant isolate harbored the A2059G mutation in the 23S rRNA. However, no resistance genes or mutations were found in one resistant isolate, suggesting an uncharacterized mechanism contributing to macrolide resistance in *G. parasuis*. Amino acid substitutions in ribosomal proteins L4 and L22 were observed in both resistant and susceptible isolates. Therefore, these substitutions alone may not be responsible for the high level of ERY resistance.

A small number of isolates showed resistance to FFC, CHL, and AMX, which agrees with previous findings (Zhou et al., 2010). The resistance genes *floR*, *catA3*, and *bla*_ROB–1_ were responsible for the resistance to FFC (*floR*), CHL (*catA3* and *floR*), and AMX (*bla*_ROB–1_), which has also been observed in previous studies (San Millan et al., 2007; Li et al., 2015).

Although this study presents some credible findings, its limitations must be considered. First, resistance breakpoints used in the present study were not species-specific but were obtained from previous reports that largely referred to other porcine respiratory bacterial species in the Clinical and Laboratory Standards Institute (CLSI) standards. Nevertheless, the lack of a *G. parasuis*-specific resistance breakpoint partly confirmed the strength of our findings. Second, we did not obtain sequencing data from the susceptible isolates (isolates susceptible to all the drugs tested). However, as the main aim of this study was to detect the underlying resistance mechanisms in resistant isolates, we prioritized sequencing the resistant group. In our future work, we aim to sequence the susceptible group.

In conclusion, *G. parasuis* isolates showed susceptibility to most of the seven antimicrobials tested. The present study demonstrated a clear association between phenotypic and genotypic resistance and revealed the heterogeneity and instability of *G. parasuis*. Our findings highlight the WGS as a valuable tool for fighting AMR and will be useful for establishing the *G. parasuis*-specific clinical breakpoints.

## Data Availability

WGS data included in this study were submitted to NCBI under BioProject accession number 279 PRJNA1184024.

## Footnotes

1
https://github.com/marbl/parsnp

2
https://itol.embl.de/

3
https://pubmlst.org/organisms/glaesserella-parasuis

4
https://pubmlst.org/bigsdb?db=pubmlst_hparasuis_seqdef&page=plugin&name=burst

5
http://genepi.food.dtu.dk/resfinder

6
http://www.ncbi.nlm.nih.gov/blast/
